# A Case of Reconstruction of an Open Tibial Fracture (Gustilo-Anderson Type IIIB) With Severe Skin and Bone Loss Using Corticocancellous Bone Plugs From the Iliac Crest and an Ilizarov Frame

**DOI:** 10.7759/cureus.22549

**Published:** 2022-02-23

**Authors:** Spyridon Papagiannis, George Sinos, Ioannis Vrachnis, Stavros Balasis, Antonis Kouzelis

**Affiliations:** 1 Orthopaedics and Traumatology, University Teaching Hospital of Patras, Patras, GRC; 2 Plastic Surgery, University Teaching Hospital of Patras, Patras, GRC

**Keywords:** corticocancellous bone plugs, orthopedic trauma, bone and skin loss, mosaicplasty bone harvesting technique, ilizarov method, sural fasciocutaneous flap, open tibial fracture

## Abstract

Gustilo-Anderson type IIIB fractures include open fractures with extensive soft tissue injury with periosteal stripping and bony exposure. They are usually associated with massive contamination and can be challenging even for experienced surgeons. A multidisciplinary approach among plastic and trauma surgeons is often required. We present a case of a 58-year-old man with a type IIIB open tibial fracture initially managed with a bridging external fixation and primary skin closure using a fasciocutaneous sural flap. Two months later, there was no evidence of fracture healing and an Ilizarov device was applied with corticotomy at the proximal tibial metaphysis, which was modified five months later without changing the frame, placing autogenous iliac bone plugs at the fracture site using the mosaicplasty harvesting technique. Seven months after its initial placement, the Ilizarov device was removed allowing full weight-bearing, with callus formation present at 10-month follow-up. Finally, the patient showed acceptable radiological and functional outcomes after a follow-up of two years. The Ilizarov method should be considered as a therapeutic option for complicated open fractures with severe bone and skin loss. The patient should be fully informed about the complexity of these fractures and the necessity of multiple surgical interventions in order to have realistic expectations.

## Introduction

Open fractures of the tibia are the most common open long bone fractures, with an annual incidence of 3.4 per 100 000, most frequently occurring in young adult males and elderly females [1]. High energy trauma is the primary mechanism of injury, with over 50% of cases being attributed to road traffic accidents or falls from a great height [1]. Noteworthy, the majority of distal tibial fractures present with a significant soft-tissue injury and therefore pose additional complicity when managing the injury, suggesting that severe open tibial fractures should be referred directly to special centers for simultaneous combined management by orthopedic and plastic surgeons [2]. The Gustilo-Anderson classification has been the mainstay of open fracture classification since it was first described in 1976. Gustilo described three broad categories, I-III, based on the extent of soft tissue injury and the size of corresponding skin wounds and then was modified in 1984 to reclassify type III fractures [3-4]. The Ganga Hospital classification for severity was developed in an effort to better prognosticate limb salvage in open tibial fractures, being of particular value in the assessment of Gustilo III-B fractures. A threshold score of 14 (out of 29) has shown good specificity and sensitivity in predicting salvage in open tibial fractures whereas a score of 17 has shown similar efficacy in predicting amputation [5]. Our aim is to present a case of a 58-year-old man with a type IIIB open tibial fracture as a result of a motorbike injury. Successful management was achieved with the Ilizarov method for both bone and soft tissue reconstruction and lengthening in different planes, combined with vascularized sural fasciocutaneous flap, free skin flap, and corticocancellous bone plugs from the iliac crest harvested using the mosaicplasty technique.

## Case presentation

**Figure 1:**
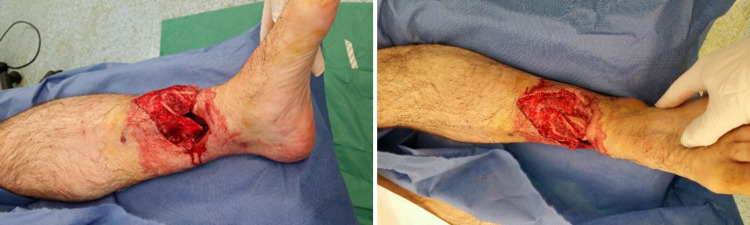
Gustilo-Anderson IIIB distal tibial fracture at the A&E department. A&E: accident and emergency

**Figure 2:**
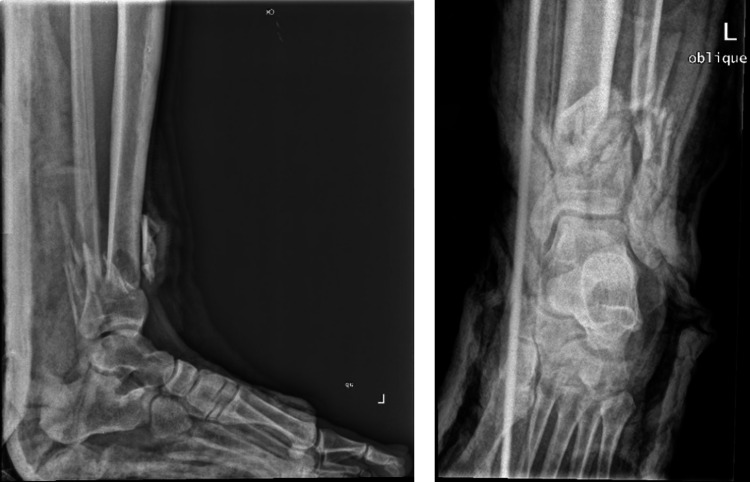
Initial anteroposterior and lateral x-rays showing excessive comminution at the fracture site.

The patient, a 58-year-old man, was transferred to the accident and emergency (A&E) department of our hospital after a motorbike injury of his left tibia. He was an otherwise healthy man with a history of smoking. He was initially evaluated according to the Advanced Trauma Life Support (ATLS) guidelines. There were no other severe or life-threatening injuries. Neurovascular status was found to be intact. There was minor bleeding at the trauma site that was successfully controlled by direct pressure. An initial intravenous antibiotic therapy was administered within three hours from the injury, consisting of a third-generation cephalosporine and metronidazole according to our hospital guidelines. The patient was also covered with tetanus prophylaxis. The limb was temporarily splinted and the patient was transferred to the radiology department. A comminuted distal tibial fracture with bone loss and a fracture of the fibula at the same level were revealed. The fracture was classified as a 43A3 according to the Arbeitsgemeinschaft für Osteosynthesefragen (AO) classification and categorized as a Gustilo-Anderson IIIB open fracture (Figure 1, Figure 2). The Ganga Hospital severity score was 10.

**Figure 3:**
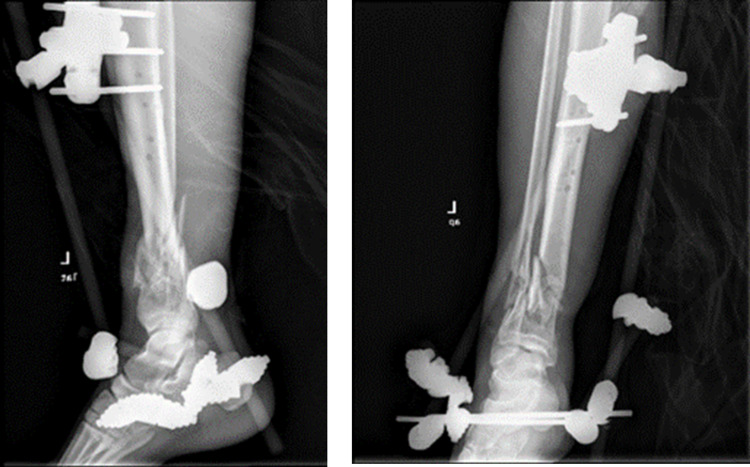
Post-operative x-rays revealing bone loss of distal tibia.

**Figure 4:**
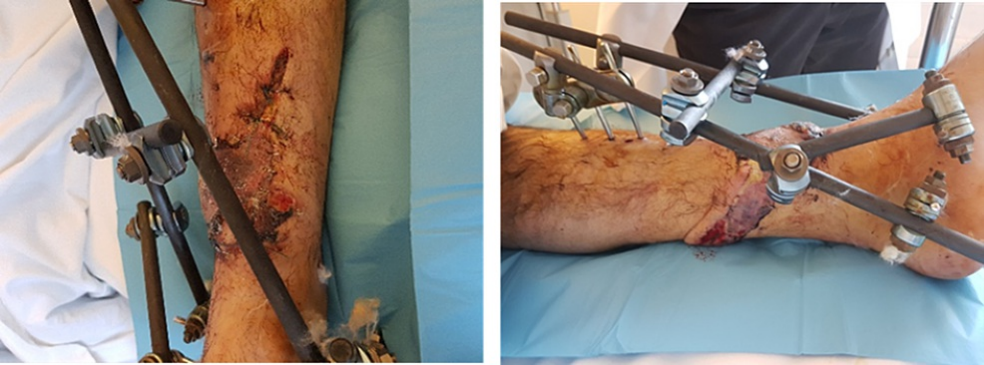
Post-operative images after the application of the sural flap.

The patient was prepped and transferred to the operating room. Under general anesthesia, thorough debridement and irrigation of the trauma were performed and a bridging external fixation with a transcalcaneal pin was applied under fluoroscopy to stabilize the fracture site temporarily (Figure 3). The skin loss at the fracture site after the initial debridement was about 8x5 cm and was temporarily covered with sterile gauzes. One week after the first procedure, the patient was transferred again to the operating room for reconstruction of the skin loss using a reverse vascularized fasciocutaneous sural flap (Figure 4). The donor site skin loss was covered using a split-thickness skin graft (STSG) from the contralateral thigh. After an uncomplicated resuscitation, the patient was transferred to the orthopedics ward. The intravenous antibiotic therapy was continued and low molecular weight heparin (LMWH) with tinzaparin 3500 IU was administered. There were no further complications during the patient’s hospitalization and the patient was discharged 27 days after his admission. Despite the extensive comminution at the fracture site, the reduction was acceptable and the initial plan was to use the external fixation as the final treatment method in order to avoid further compromising the soft tissue envelop and endangering the viability of the previously applied flap.

**Figure 5:**
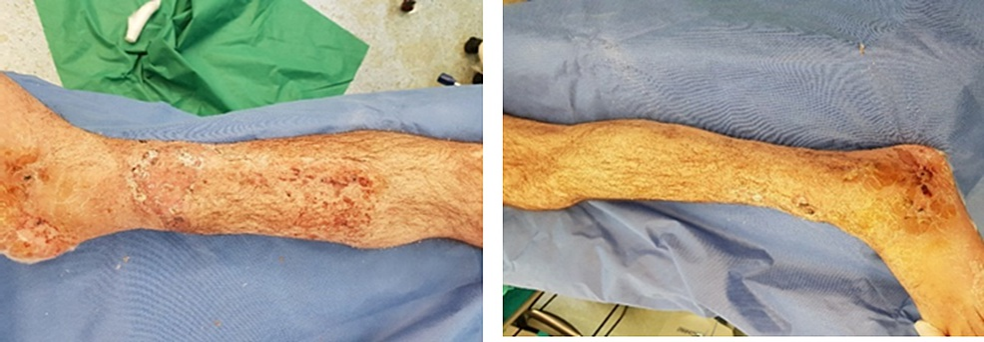
Two-month follow-up. Healing of the flap.

**Figure 6:**
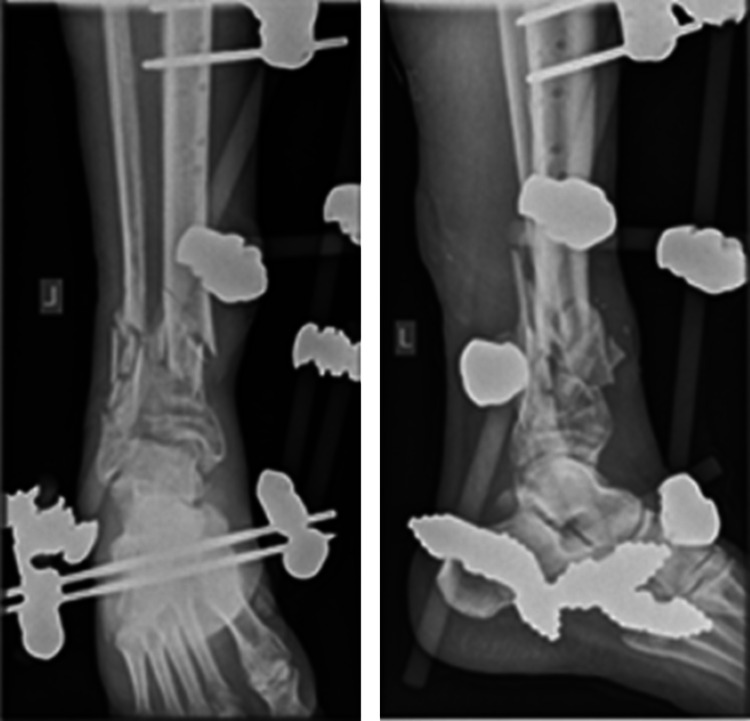
Two-month follow-up. Delayed union at fracture site.

At the two-month follow-up, great integration and healing of the flap were identified (Figure 5); however, there was no union progress at the radiological control (Figure 6). The patient was re-admitted to the orthopedic department, the external fixation was removed and he was rescheduled for a new surgical intervention. Pin-site cultures were negative and the erythrocyte sedimentation rate (ESR) and C-reactive protein (CRP) levels were normal. Although the two-month period is not a long time to expect fracture healing, the decision was made to proceed to the application of the Ilizarov frame in order to progressively enhance callus formation through distraction osteogenesis.

**Figure 7:**
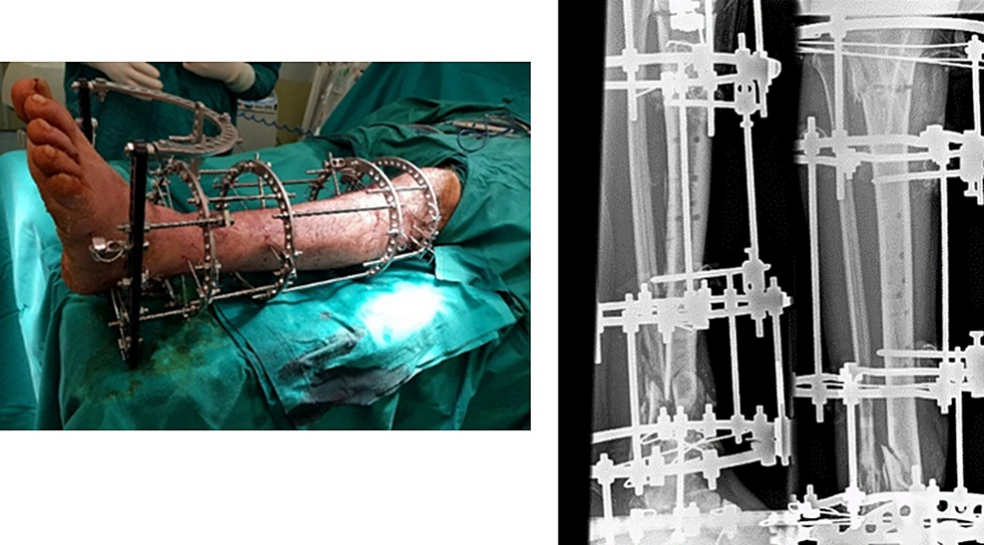
Intraoperative image and post-operative x-rays of the Ilizarov technique.

Three months after the initial injury the patient was transferred to the operating room where an Ilizarov device was applied under general anesthesia and fluoroscopy (Figure 7). Three rings were applied proximal and one ring distal to the fragment site and a footplate as well. Low energy osteotomy was performed between the two proximal rings. The Ilizarov method was considered to be the most suitable treatment option since it can provide the ability to reconstruct the bone defect using distraction osteogenesis without periosteal stripping, thus not compromising the biological environment that could enhance the healing potential. Moreover, based on the fact that it was a closed procedure, minimum damage would be added to an already compromised soft-tissue envelope. Bone transport was initiated 10 days postoperatively at a rate of 0.75 mm/d [6-7]. This rate had to be reduced when pain was not well tolerated [8]. Partial weight-bearing was allowed two weeks after the operation.

**Figure 8:**
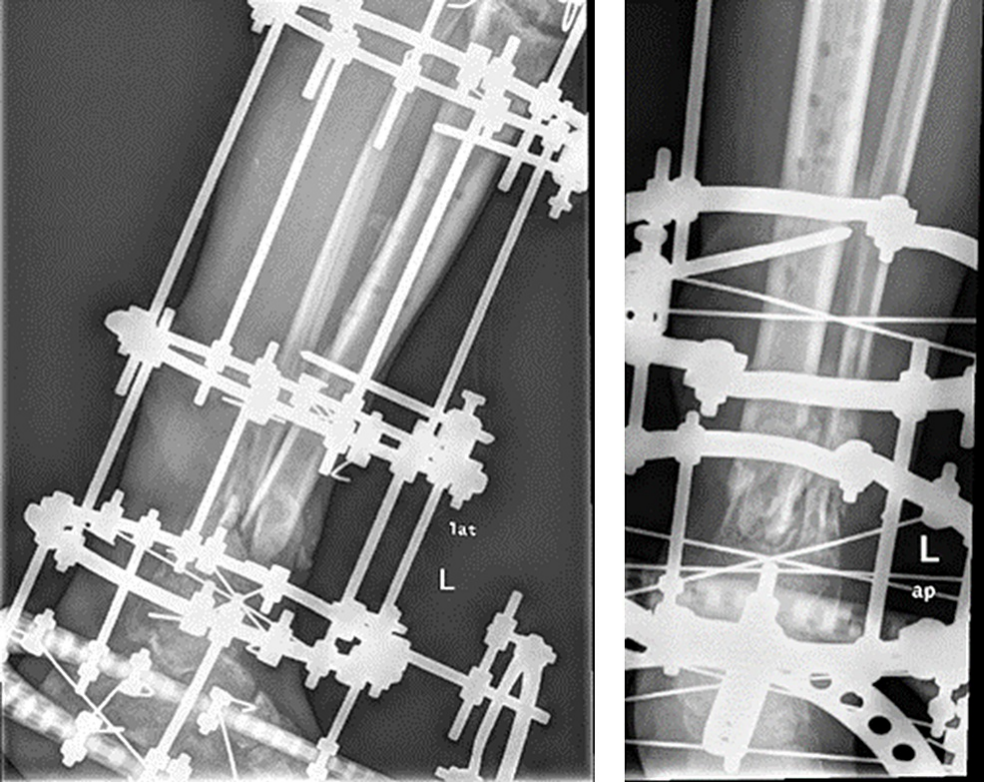
Nonunion still present at the eight-month follow-up.

**Figure 9:**
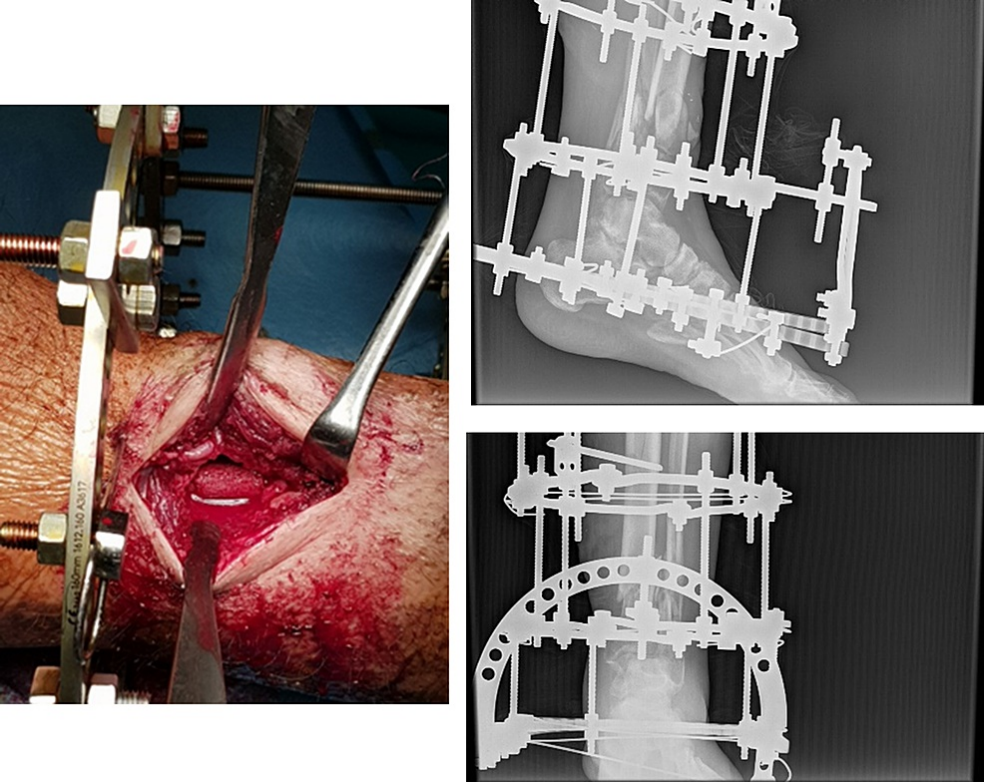
Iliac autografts cylinders placement and post-operative x-rays.

During the follow-up period, nonunion at the fracture site was still present eight months after the initial injury and five months after the Ilizarov device application (Figure 8). The patient was admitted again to the orthopedic department and a new surgery was scheduled. Under general anesthesia, iliac corticocancellous autograft plugs were harvested using the mosaicplasty instrumentation and placed at the nonunion site (Figure 9). Meanwhile, the Ilizarov device was modified in order to achieve acceptable reduction and compression at the area. Ten months after the initial injury, signs of callus formation were present at the fracture site.

**Figure 10:**
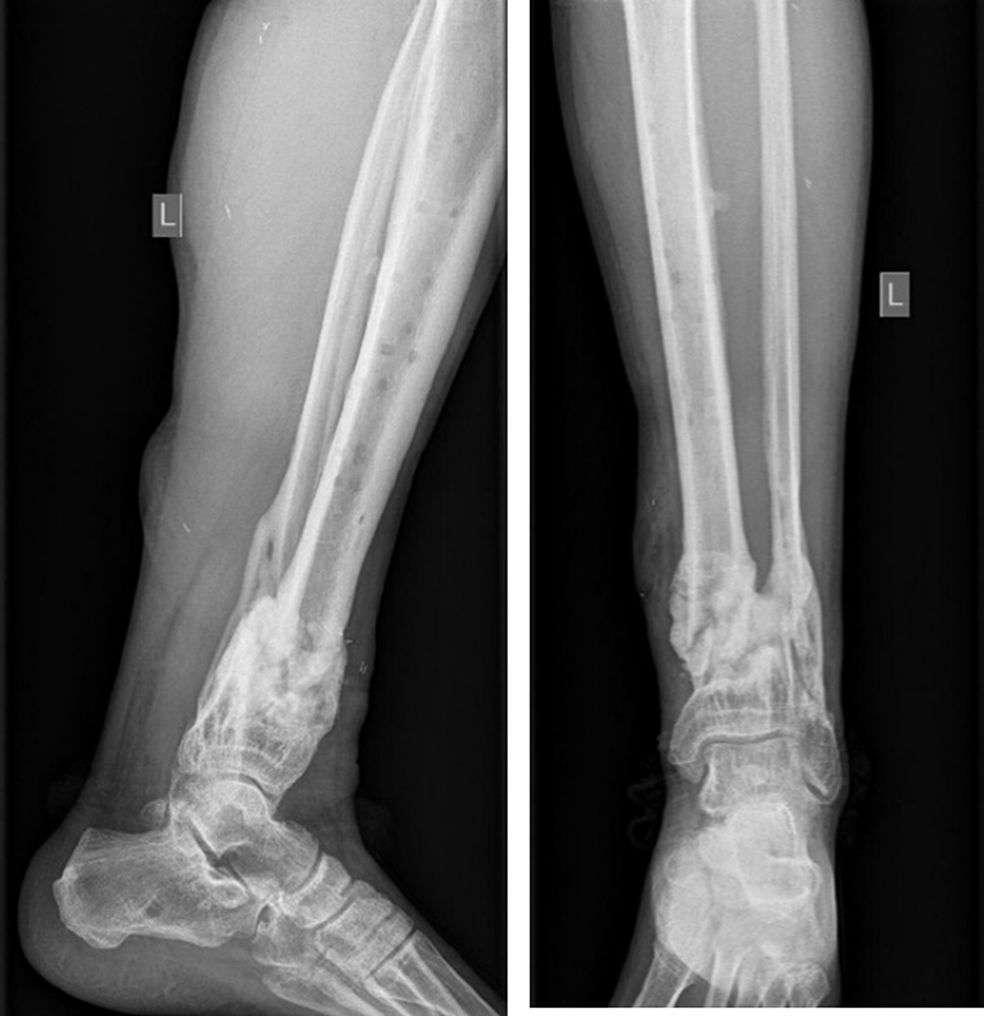
14-month follow-up. Callus formation is present.

**Figure 11:**
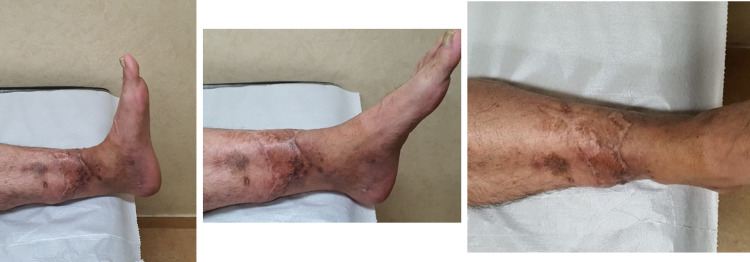
24-month follow-up. Range of motion at the ankle joint.

Finally, at the 10-month follow-up and seven months after the initial placement, the Ilizarov device was removed and partial weight-bearing was suggested. At the 14-month follow-up, callus formation was established (Figure 10). At the two-year follow-up, mild restriction of movement at the ankle joint was present (Figure 11) and the patient was able to walk without crutches. The American Orthopedic Foot and Ankle Score (AOFAS) score at that time was 79%.

## Discussion

Open fractures of the distal tibia complicated with severe soft tissue injuries present a challenge for accomplishing both fracture union and wound healing [9]. Distraction osteogenesis using the Ilizarov method can maintain the reduction of fractures or nonunions, stimulate bone formation, eliminate the need for implanted hardware, provide a stable platform for soft tissue reconstruction, and allow full weight-bearing [10-11]. Jitprapaikulsarn et al. described the simultaneous use of internal fixation using plates and screws and soft tissue coverage by a distally-based sural flap for complex Gustilo IIIB open fractures as an effective and reproducible technique [12]. To date, the role of distally-based fasciocutaneous flaps in concurrent ring external fixation using the Ilizarov method and soft tissue coverage is still not specifically described.

Hu et al. investigated the application of local flaps and Ilizarov osteogenesis in the reestablishment of severe combined defects of tibial bone and soft tissue. In a series of 16 patients, all flaps survived and 15 patients healed with no limb-length discrepancy [13]. The sural pedicled flap constitutes a well-vascularized cutaneous islet and reliable flap offering the possibility of covering a broad range of areas with cutaneous defects in the distal tibia. It requires a one-stage operation with no need for microsurgical instrumentation or anastomosis and without compromising the vascularization of the lower limb, thus making it a simple and reproducible procedure [14]. However, many complications have been associated with the use of local fasciocutaneous flaps, with necrosis, failure of the intent, and donor-site problems being the most common [15]. The use of local fasciocutaneous flaps requires that the relationship between the fascia and the local tissues proposed for transfer is not predisposed in any way. There are several concerns regarding the concomitant application of the Ilizarov device and local fasciocutaneous flaps. Pin insertion and postoperative distraction can jeopardize the viability of the pedicle by distorted local anatomy due to the deposition of scar tissue as well as local vascular spasm. Great care must be taken by the orthopedic team to the exact course of the pedicle before pin insertion to avoid vessel injury. In the case presented the perforating branches of the peroneal vascular trunk withstood not only the severity of the initial injury but also changes to the limb length caused by the placement of the Ilizarov device. This may be due to the stabilization of soft tissue reconstruction, the small diameter of the wires, the atraumatic technique of percutaneous placement used, and the use of tolerable distraction rate. Nonetheless, parameters such as the cost of treatment, the complexity of additional surgery, and the duration of the whole treatment should also be considered [16].

Autogenous bone graft remains a reliable treatment option providing an osteoconductive, osteoinductive, and osteogenic substrate for filling bone voids and augmenting fracture-healing, with the iliac crest being the most frequently used site for bone-graft harvest [17]. The most common complication associated with the harvest of autogenous bone graft is pain at the donor site, with less frequent complications including nerve injury, hematoma, infection, and fracture at the donor site [18]. Many techniques have been described for iliac autograft harvesting. In our case, we used the mosaicplasty instrumentation to obtain good quality corticocancellous cylinders, an autograft harvesting technique described as having fewer donor site complications and higher patient satisfaction rates [19]. Autologous bone grafting is indicated for defects less than 5cm [20] with a well-vascularized, healthy recipient site. In cases of larger bone defects, the induced membrane (Masquelet) technique can be used with good results compared with distraction osteogenesis, taking into consideration the autograft requirements and the donor site complications. Distraction osteogenesis has been used to successfully treat large bone defects with a less invasive technique, minimizing blood loss, and with no need for grafting. However, in our case, since the inadequate union of the fracture was present after the initial placement of the device, we decided to use autologous iliac crest grafts.

## Conclusions

In our case, distraction osteogenesis with the Ilizarov technique was combined with autologous iliac crest grafting in order to achieve bone healing, while a sural fasciocutaneous flap was used in order to restore the soft tissue loss. The mosaicplasty technique can be an alternative in iliac autograft harvesting with low complication rates and good clinical results. A multidisciplinary approach may be required for the treatment of complex distal tibial fractures with both bone and soft tissue loss in order to ensure not only limb salvage but a good clinical outcome as well.
